# Relationship between HIV-1 Gag Multimerization and Membrane Binding

**DOI:** 10.3390/v14030622

**Published:** 2022-03-16

**Authors:** Christopher Sumner, Akira Ono

**Affiliations:** Department of Microbiology and Immunology, University of Michigan, Ann Arbor, MI 48109, USA; sumnerch@umich.edu

**Keywords:** HIV-1, particle assembly, Gag, multimerization, membrane binding, tRNA binding, genomic RNA binding, capsid, nucleocapsid, matrix

## Abstract

HIV-1 viral particle assembly occurs specifically at the plasma membrane and is driven primarily by the viral polyprotein Gag. Selective association of Gag with the plasma membrane is a key step in the viral assembly pathway, which is traditionally attributed to the MA domain. MA regulates specific plasma membrane binding through two primary mechanisms including: (1) specific interaction of the MA highly basic region (HBR) with the plasma membrane phospholipid phosphatidylinositol (4,5) bisphosphate [PI(4,5)P_2_], and (2) tRNA binding to the MA HBR, which prevents Gag association with non-PI(4,5)P_2_ containing membranes. Gag multimerization, driven by both CA–CA inter-protein interactions and NC-RNA binding, also plays an essential role in viral particle assembly, mediating the establishment and growth of the immature Gag lattice on the plasma membrane. In addition to these functions, the multimerization of HIV-1 Gag has also been demonstrated to enhance its membrane binding activity through the MA domain. This review provides an overview of the mechanisms regulating Gag membrane binding through the MA domain and multimerization through the CA and NC domains, and examines how these two functions are intertwined, allowing for multimerization mediated enhancement of Gag membrane binding.

## 1. Introduction

**Figure 1:**
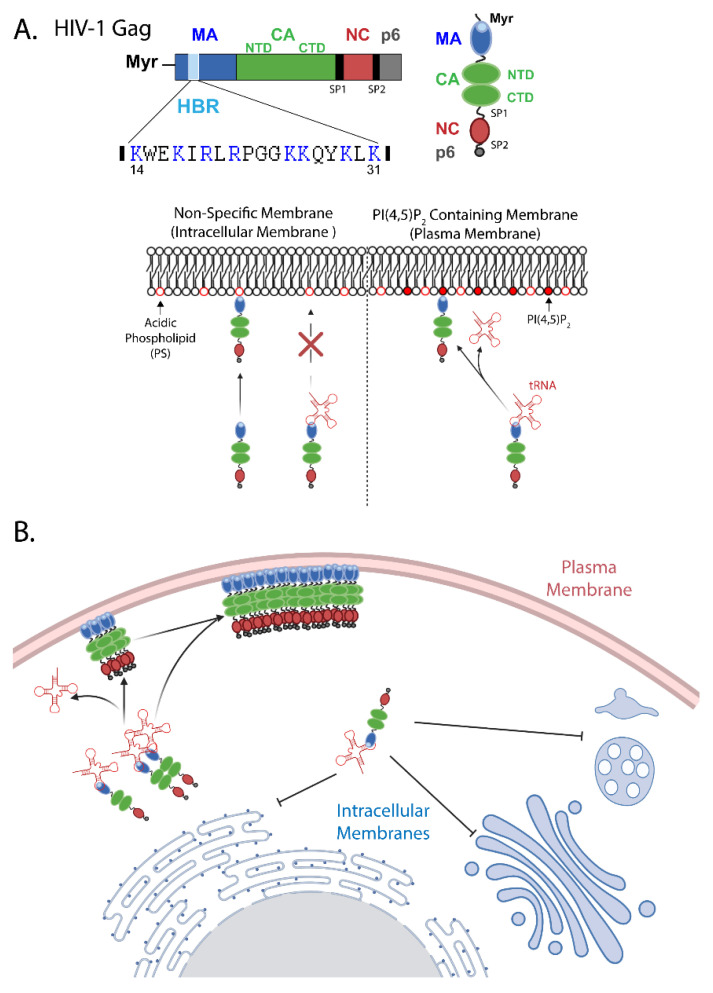
Plasma membrane-specific binding of HIV-1 Gag. (**A**) Molecular determinants for Gag membrane binding. *Top*, the domain organization of HIV-1 Gag. Blue, matrix (MA); green, capsid (CA, N-terminal domain [NTD] and C-terminal domain [CTD]); red, nucleocapsid (NC); gray, p6; and black, spacer peptides 1 and 2 (SP1 and SP2). Additionally, the locations of the myristoyl moiety (Myr) and the highly basic region (HBR, light blue) are shown. The HBR amino acid sequence is shown with basic residues highlighted in blue. *Bottom*, host molecules that bind the MA HBR. Gag binds membranes through interactions between the HBR and acidic phospholipid headgroups. tRNA binding to the HBR prevents interaction with membranes that contain a prevalent acidic phospholipid, phosphatidylserine (PS), but allows for Gag binding to membranes containing phosphatidylinositol (4,5) bisphosphate [PI(4,5)P_2_]. For clarity, the myristoyl moiety is not depicted. (**B**) A model that accounts for specific localization of Gag to the plasma membrane. tRNA-mediated inhibition of membrane binding prevents localization of Gag to intracellular membranes, which lack PI(4,5)P_2_, but allows for binding to the plasma membrane where PI(4,5)P_2_ is present.

During the HIV-1 replication cycle, the processes of viral particle assembly and release are driven primarily by the structural protein Gag. The 55kDa Gag precursor polyprotein Pr55Gag contains matrix (MA), capsid (CA), nucleocapsid (NC), and p6 domains as well as two spacer peptides (SP1 and SP2) (Figure 1A top). Viral assembly begins with translation of the Gag protein from the unspliced viral mRNA in the cytoplasm of the infected cell. After translation, the Gag protein accumulates at the plasma membrane of the host cell, driven by the specific interaction of the MA domain with the plasma membrane phospholipid phosphatidylinositol (4,5) bisphosphate [PI(4,5)P_2_]. Once Gag binds to the plasma membrane, inter-Gag interactions mediated by CA-CA and NC-RNA binding drive the formation of the immature lattice at the site of viral particle assembly. Here, viral genomic RNA (gRNA) is selectively packaged through the specific interaction of the packaging element (psi) with the NC domain of Gag, ensuring incorporation into the growing viral particle. Through addition of Gag monomers and dimers, this lattice grows, inducing membrane curvature at the site of viral assembly and the budding of the immature viral particle from the surface of the host cell. The host cell ESCRT machinery is recruited to the site of viral assembly by the p6 domain of Gag, which is responsible for the scission of the membranous neck and the release of the immature viral particle (HIV-1 particle assembly is reviewed in [1]). In this review, we will summarize current knowledge regarding the viral and cellular determinants that regulate two essential steps of HIV-1 particle assembly, i.e., Gag membrane binding and multimerization, and then discuss the relationships between the two steps as well as questions that remain to be answered.

## 2. Mechanisms Regulating HIV-1 Gag Membrane Binding

The membrane binding activity of HIV-1 Gag during assembly is attributed to the MA domain. The HIV-1 MA domain contains two major membrane binding signals, an N-terminal myristoylation (Myr) and a conserved stretch of basic amino acids [2,3,4,5,6,7,8,9,10] (Figure 1A top).

### 2.1. The Role Played by N-Terminal Myristate Moiety

The myristate moiety, which is added to the N-terminal glycine of the MA domain after the removal of the first methionine, contributes to MA membrane binding through hydrophobic interactions with the membrane bilayer. Substitutions of the MA N-terminal residues that prevent the acylation by myristoyl transferase drastically reduces the membrane binding of Gag and the production of viral particles [2,3,5,8,11,12]. While myristate is often found on membrane binding proteins, this modification alone is not expected to drive selective binding to the plasma membrane (reviewed in [13,14]).

### 2.2. The Role Played by the Highly Basic Region

In addition to myristoylation, early work determined that a polybasic sequence spanning MA residues 14–31 is also involved in membrane binding [9,10,15]. This highly basic region (HBR) forms a positively charged patch on the surface of the MA globular domain [15,16,17,18] and mediates membrane binding through electrostatic interactions with the acidic headgroups of phospholipids such as phosphatidylserine (PS) [5,19,20,21,22].

### 2.3. PI(4,5)P_2_ Binding and Plasma Membrane Specificity

HIV-1 particle assembly takes place at the plasma membrane [23,24]. Cell-based and in vitro studies have collectively demonstrated that a specific interaction of the MA HBR with the phospholipid phosphatidylinositol (4,5) bisphosphate [PI(4,5)P_2_], which is present specifically in the inner leaflet of the plasma membrane, drives the selective binding of Gag to the plasma membrane in the host cell and hence particle assembly at this location [6,23,25,26,27,28,29]. When a phosphatase that depletes PI(4,5)P_2_ is ectopically expressed in HIV-expressing cells, Gag fails to bind membrane efficiently, resulting in a primarily cytosolic distribution, with a subset of cells displaying mislocalization of Gag to internal compartments instead of the plasma membrane. Consequently, HIV-1 particle release is drastically reduced in these cells. Suppression of host enzymes involved in PI(4,5)P_2_ generation has a similar effect [30,31]. Interestingly, induction of PI(4,5)P_2_-enriched endosomal structures also causes a defect in the specific localization, i.e., Gag accumulation at these intracellular vesicles, and a reduction in viral particle release [6].

PI(4,5)P_2_ is important not only for specific targeting of Gag to the plasma membrane, but also for stable association of Gag with this membrane once bound. Using a system that allows reversible depletion of PI(4,5)P_2_ at the plasma membrane, Mücksch et al. demonstrated that pre-assembled Gag lattices are detached from the plasma membrane upon depletion of PI(4,5)P_2_, disassembling back into monomers or lower level oligomers which remain assembly competent [32]. This suggests that even after the establishment of the immature lattice on the membrane, the HBR-PI(4,5)P_2_ interaction is required for the maintenance of the lattice, preventing the equilibrium of Gag membrane binding from shifting to a cytosolic distribution [32].

In vitro experiments demonstrate that Gag binds more efficiently to membranes containing PI(4,5)P_2_ than those containing other acidic phospholipids such as PS [25,33,34,35,36,37,38,39,40,41,42]. Increasing the proportion of non-PI(4,5)P_2_ acidic phospholipids to compensate for the greater negative charge of PI(4,5)P_2_ does not result in a similar increase in Gag membrane binding, suggesting that Gag recognition of PI(4,5)P_2_-containing membranes is not simply charge-based but likely involves some recognition of the PI(4,5)P_2_ headgroup structure [25,38,39,40]. Multiple studies have shown that the interaction of Gag with highly acidic head group of PI(4,5)P_2_ involves residues within the HBR [25,26,27,43]. This suggests that while MA HBR can mediate membrane binding through interaction with any acidic phospholipid (such as PS), specific recognition of the PI(4,5)P_2_ headgroup by residues within the HBR preferentially drives high affinity interaction of Gag with PI(4,5)P_2_-containing membranes.

### 2.4. RNA-Mediated Suppression of MA-Lipid Binding

In addition to acidic phospholipids, MA has also been shown to bind nucleic acids [27,34,35,44,45,46,47,48,49,50]. RNA binding to the MA domain involves regions that overlap with the site of PI(4,5)P_2_ interaction within the HBR [44,51,52,53,54]. While the RNA interaction with the HBR inhibits Gag binding to membranes containing non-PI(4,5)P_2_ acidic phospholipids such as PS, in the absence of RNA, Gag binds these membranes efficiently (Figure 1A bottom). Even in the presence of RNA, Gag retains the ability to bind membranes containing PI(4,5)P_2_ efficiently, suggesting that PI(4,5)P_2_ can out-compete RNA for binding to the MA HBR. It is important to note that even in the presence of PI(4,5)P_2_, the presence of RNA can partially inhibit Gag membrane binding, suggesting that the effect of RNA on Gag-acidic lipid interactions is not binary [25,34,35,55]. Together, these results suggest a hierarchy in which HIV-1 MA HBR binds to PI(4,5)P_2_ more strongly than RNA, which binds more strongly than other acidic phospholipids such as PS. Thus, while RNA binding to the MA HBR shields it from non-specific interaction with acidic phospholipid containing membranes, PI(4,5)P_2_ overcomes RNA-mediated inhibition and allows for efficient Gag membrane binding (Figure 1A bottom).

In cells, an RNA crosslinking study showed that tRNA was the major RNA species that binds HIV-1 MA in the cytosol [56]. In vitro experiments demonstrated that tRNA can block Gag binding to non-PI(4,5)P_2_-containing membranes at concentrations below what is present in the cytosol [42,55]. These observations suggest that in the cellular context tRNA is primarily responsible for the negative regulation of Gag membrane binding, preventing Gag association with intracellular membranes that lack PI(4,5)P_2_, while allowing for Gag accumulation specifically at the plasma membrane where PI(4,5)P_2_ is present (Figure 1B). This model is supported by cellular data demonstrating that mutations disrupting MA-RNA binding (K25, K26 and K29, K31 mutants) cause defects in the specific localization of Gag to the plasma membrane [53]. Additionally, HIV-1 Gag chimeras containing MA domains from other retroviruses that are sensitive to RNA-mediated inhibition localize specifically to the plasma membrane, while Gag containing RNA-insensitive MA domains localize promiscuously to intracellular membranes [33,37]. While these studies suggest that tRNA binding to MA HBR spatially regulates Gag membrane binding, it is also conceivable that tRNA imparts some temporal regulation, for example, by preventing pre-mature plasma membrane binding when Gag concentration is still low [57]. Interestingly, although full-length Gag overcomes tRNA-mediated inhibition and binds membranes if they contain PI(4,5)P_2_ [34], isolated MA domains do not efficiently bind PI(4,5)P_2_-containing membranes in the presence of tRNA [52]. These observations support the possibility that, in addition to the competition between PI(4,5)P_2_ and tRNA for binding to the basic residues of the HBR, the Gag domains downstream of MA play a role in regulating tRNA displacement from MA HBR.

## 3. CA-CA Interactions and Their Role in Immature Gag Lattice Structure

**Figure 2:**
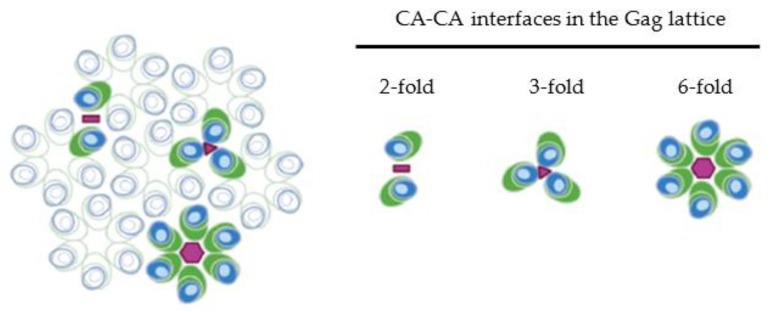
Organization of MA and CA in the membrane-bound Gag lattice. Gag domains are depicted as in Figure 1 but only MA and CA are shown. Blue, MA; light blue, HBR; and green, CA. Three types of CA-CA interactions contributing to formation of immature Gag lattice are shown on the right. The two-fold, three-fold, and six-fold interaction interfaces are denoted by pink bar, triangle, and hexagon, respectively. Six-fold interactions involving the MHR and six-helix bundle (formed by CA-CTD and SP1 regions) form Gag hexamer subunits, with interactions at the two-fold and two-fold interfaces binding adjacent hexamer rings to one another. In the immature lattice, MA trimers form above the two-fold CA interaction interface, with an MA domain presumably contributed from each of three adjacent hexamer rings.

The interactions between adjacent CA domains of the HIV-1 Gag polyprotein are the driving force behind the viral assembly and the organization of the immature hexagonal Gag lattice. HIV-1 CA contains two sub-domains, the N-terminal domain (NTD) and the C-terminal domain (CTD), which play distinct roles in the immature lattice. Both the major homology region (MHR) within the CA CTD [58,59,60,61,62] and a six-helix bundle formed by the region spanning the C terminus of the CA CTD and the N terminus of SP1 contribute to the six-fold interaction interface at the center of the CA hexameric ring [63,64,65,66,67,68,69]. X-ray structures reveal that the cellular co-factor inositol hexakisphosphate (IP_6_) binds two rings of lysine residues at positions 158 and 227 of the HIV-1 CA domain, stabilizing the six-helix bundle in the immature Gag lattice, and mutations in these residues disrupt CA-interactions at this interface [70,71,72]. In addition to the six-fold interactions, residues within the CTD mediate the two-fold interface between CA domains [60,62,63,73,74]. Mutations within the CA-NTD (such as CA P38E, A42E, E45R) also cause defects in virus assembly [39,73], and residues within this sub-domain contribute to the three-fold CA interaction [62,75]. Together, the CA-NTD, CA-CTD, and SP1 regions cooperate to form an immature Gag lattice consisting of hexametric rings that assemble with one another at their two- and three-fold interfaces (illustrated in Figure 2).

## 4. Enhancement of Gag Multimerization by NC-RNA Interaction

While the NC domain of HIV-1 Gag promotes selective packaging of the genomic RNA (gRNA) through its specific interaction with the psi element (reviewed in [76,77,78]), the RNA binding capacity of the NC domain also promotes efficient assembly of the immature lattice. The presence of nucleic acids promotes efficient in vitro assembly of Gag [79,80,81,82,83]. In cells, deletion or substitution of basic arginine and lysine residues within NC, which reduces NC-RNA binding, disrupts Gag–Gag interaction and particle assembly/release [84,85]. Together, these findings suggest that non-specific RNA binding by NC basic residues contribute to Gag multimerization and efficient particle assembly. Multiple studies have demonstrated that replacement of NC with heterologous domains mediating protein–protein interaction allows for efficient particle production regardless of the ability to bind RNA [86,87,88,89,90], indicating that the specific NC-RNA interaction is not required for efficient Gag multimerization. In the absence of packageable gRNA, full length HIV-1 Gag encapsidates cellular RNA species non-selectively [91], suggesting that non-specific RNA binding can also play a role in enhancing Gag assembly in the cellular environment.

It is likely that NC-RNA interactions enhance Gag multimerization through scaffolding of Gag assembly, with nucleic acids bridging multiple adjacent NC domains, facilitating inter-CA interactions. This effect has been observed in in vitro assembly reactions with short oligonucleotides (10–15 bases) promoting Gag assembly [81]. A similar effect is seen with RSV Gag, where short oligonucleotides (16 bases) promote assembly through dimerization of the Gag polyprotein [92,93]. In the context of Gag assembly in cells, single particle tracking PALM (photoactivated localization microscopy) and coarse grained computational modeling data suggest that longer gRNA serves as a scaffolding for Gag assembly, regulating the rate of Gag protein incorporation into the immature lattice as well as the recruitment and tethering of Gag assembly precursors to the membrane at the site of viral assembly [94,95]. Additionally, it has been observed that even extremely short oligonucleotides, which cannot bind multiple NC domains, may nucleate Gag assembly through the formation of nucleic acid-triggered NC-dimers, which enhance Gag dimerization more efficiently than CA-CA interactions [81,96].

In addition to these general mechanisms mediated by any RNA, specific interaction of Gag with packageable viral gRNA also contribute to efficient Gag multimerization. In cells, both Gag-viral gRNA interaction in the cytoplasm and trafficking of the gRNA to the plasma membrane are dependent on interactions with two zinc finger motifs, which play partially redundant roles [97]. Particle production is enhanced by the presence of packageable viral RNA in a manner reliant on specific recognition of packageable RNA by the Gag NC domain [98]. In vitro experiments demonstrate that Gag assembly is more efficient in the presence of HIV RNA containing clusters of unpaired G residues within the nucleocapsid interaction domain of the psi sequence (identified in [99]) compared to RNA constructs lacking these clusters [100]. Additionally, if assembly is seeded with complexes capable of high affinity NC-psi interaction, addition of bulk Gag that lacks specific high affinity interaction is sufficient for efficient growth of the immature lattice [101]. This suggests that high affinity NC-psi interaction is only required for the formation of the initial Gag complex but is not necessary for lattice growth. Additionally, NC domains in the context of full length Gag show greater affinity for psi containing RNA, forming “early complexes” in association with 5′ leader sequence which are dependent on CA dimerization [102]. These data are consistent with a recently proposed model where specific NC binding to the viral genomic packaging element psi promotes nucleation of the initial Gag assembly complex (reviewed in [77]). Interestingly, while many NC binding sites have been identified within the HIV-1 genomic leader sequence, a subset of non-electrostatic high affinity binding sites exist at a GGAG sequence that is associated with a three-way junction within the dimerized leader [103,104]. Binding of NC at these locations triggers re-modeling of the RNA structure of the leader, which is essential for specific packaging of genomic RNA [103]. Based on these data, Ding et al. suggest a mechanism where initial NC binding to these high affinity sites stimulates recruitment of additional Gag molecules to the gRNA, nucleating Gag assembly in association with packageable genome.

## 5. Formation of the Gag Lattice at the Plasma Membrane and Potential Contribution of Cytosolic Gag-RNA Complexes

A previous chemical crosslinking study suggested that in the cytoplasm of the cell, Gag exists primarily as a monomer or a low order multimer [56]. In this study, much of the Gag population progresses to higher order multimerization only when it reaches the plasma membrane. This is supported by FRET experiments which demonstrate that the majority of Gag-Gag oligomerization occurs at the plasma membrane, although some FRET signal is found in the cytosol [105,106,107]. Both VLP assembly studies and image-based analysis of Gag assembly in cells by FRET or transmission electron microscopy demonstrate that MA-membrane binding and NC-RNA interaction play functionally redundant roles promoting Gag oligomerization [49,107,108]. In agreement with these results, a coarse-grained simulation model suggests that both RNA and membranes function as scaffolds promoting Gag assembly [95].

Using a photoconvertible labeling system paired with TIRF excitation, Ivanchenko et al. showed that Gag clusters that nucleate sites of viral assembly are primarily recruited from the cytoplasm [109]. Consistent with this result, quantitative measurements of Gag plasma membrane association using a combination of dual-color, z-scan florescence fluctuation spectroscopy with epifluorescence and TIRF microscopy showed that HIV-1 Gag does not bind membrane until cytosolic concentration is high. This, in turn, suggests that Gag does not bind the membrane as a monomer and, hence, is unlikely to be recruited laterally as monomers into sites of viral assembly [110].

Cytosolic complexes containing Gag proteins have been identified [111,112,113], which have been proposed to function as assembly intermediates involving both Gag and viral gRNA upstream of the establishment of the Gag lattice on the plasma membrane [114]. Gag has been shown to traffic progressively through these complexes by pulse chase labeling approaches [112,115] as well as by analysis of Gag mutants defective in different steps of multimerization [114,116]. In this proposed assembly pathway, Gag first associates with the genomic RNA in the ~80S complex, which is associated with RNA granule proteins DDX6 and ABCE1 [111]. Of note, a recent work has suggested that these smaller cytosolic complexes (up to ~80S) likely consist primarily of monomeric or dimeric Gag associated with ribosomes or other RNA-containing cellular complexes and might not represent assembly intermediates containing cytosolic Gag multimers [117].

**Figure 3:**
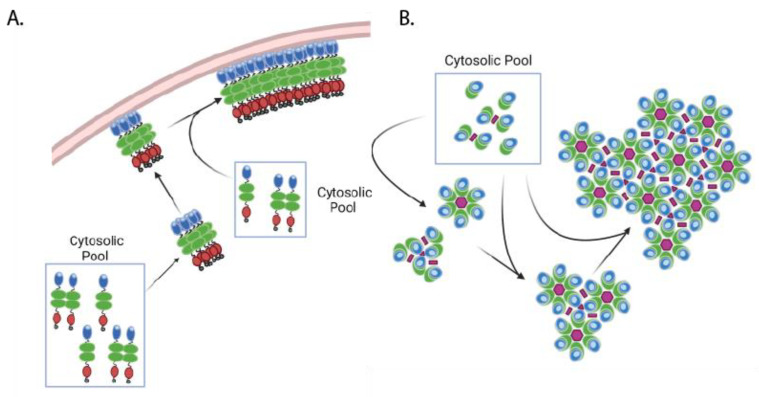
Immature lattice establishment and assembly progression. Side view (**A**) and top view (**B**) of lattice formation and growth. Initial Gag complexes arrive at the plasma membrane from the cytosol, potentially in complex with genomic RNA. Higher order multimerization occurs at the plasma membrane, with monomeric and dimeric Gag (from the cytosolic pool) added to the growing lattice edges. Gag domains are shown in the same color scheme as in Figure 1 and Figure 2. Pink polygons depict different CA-CA interfaces as described in Figure 2.

Although the identity of the “~80S intermediate” requires further refinement, this complex may play a role in Gag assembly as the cytosolic site of Gag-gRNA binding and may even nucleate lattice formation upon binding to the plasma membrane. Consistent with this model, a fluorescence fluctuation imaging study observed Gag oligomerization in the cytosol, supporting the possibility that formation of cytosolic viral gRNA-associated Gag clusters precedes immature lattice assembly [118]. Additionally, the presence of packageable gRNA dimers has been shown to enhance viral assembly [98,119]. Early experiments utilizing TIRF imaging of HIV-1 assembly sites suggest that gRNA arrives to the plasma membrane as a pre-assembled dimer complex [120], while later TIRF experiments utilizing two-color imaging observed gRNA dimerization primarily at the plasma membrane [121]. However, a 3D super-resolution microscopy using a two-color strategy revealed that dimerization of gRNA occurs in the cytosol, and Gag facilitates this process [122]. Although the spatiotemporal details of gRNA dimerization during HIV-1 assembly are still unclear, it is conceivable that gRNA-associated Gag clusters at the plasma membrane “seed” assembly of the immature Gag lattice [77,100,119,123] (Figure 3A). Consistent with this possibility, recently published work demonstrated that mutations disrupting Gag dimerization (CA W184A, W185A) or membrane binding (MA 1GA) introduced in Gag molecules bound to viral RNA impair viral RNA packaging even when these mutant Gag molecules can coassemble into particles formed by assembly competent but RNA-binding-incompetent Gag that is coexpressed [124]. Because the initial Gag-gRNA complex likely plays a key role in the establishment of the immature lattice at the site of assembly and ensures efficient viral RNA packaging, more in-depth study of when this complex is established and how it is incorporated specifically into the assembly site at the plasma membrane is needed.

Single particle tracking data indicate that after the nucleation of assembly on the membrane, Gag is primarily added to the growing immature lattice as lower order multimers such as dimer [94] (Figure 3B) [94]. In agreement with these findings, in silico modeling of lattice growth and measurement of membrane-bound lattice dynamics by atomic force microscopy demonstrate that the immature Gag lattice grows through the addition of monomer and dimer subunits and not by the addition of completed hexamer rings [125,126]. Together, these results suggest a model for HIV-1 particle assembly where the arrival of a Gag complex, which likely contains viral gRNA, from the cytosol to the plasma membrane nucleates Gag lattice formation, which is followed by its growth driven by the recruitment of monomeric or dimeric Gag from the cytosolic pool.

## 6. Organization of MA in Membrane-Bound Gag Multimer

While the MA-MA interaction is generally not thought to contribute to the overall oligomerization of Gag or formation of the Gag lattice, the MA domain also multimerizes into trimers within the immature Gag lattice. MA domains have been observed to form trimers both in crystal structures and in solution [15,127,128]. Studies utilizing transmission electron microscopy analysis of membrane-bound protein crystals demonstrate that upon binding to membranes containing phosphatidylserine (PS) MA organizes into hexamers [22]; however, on PI(4,5)P_2_-containing membranes MA domains from both purified MA and MA-CA proteins organize as trimers [36]. In agreement with these two-dimensional crystal data, recent cryo-electron tomography showed that MA within the Gag lattice of immature particles organizes in a similar hexamer of trimers arrangement [129]. Interestingly, in the context of either membrane-bound MACA protein [36] or the immature full length Gag lattice [129], the MA trimer is situated above the three-fold CA interface, suggesting that each MA domain within the trimer is contributed by a Gag molecule from three adjacent hexamer rings (Figure 2). The trimerization of the MA domain within the immature lattice is thought to play functional roles in the incorporation of the envelope glycoprotein into the assembling viral particle [130,131] and in the exposure of myristate [128], which is discussed later in more detail.

## 7. Genetic Evidence for Multimerization-Mediated Enhancement of Gag Membrane Binding

Oligomerization-competent Gag precursor shows greater affinity for model membranes than mature Gag proteins that correspond to domains of the precursor (including MA) [19]. In the context of full-length Gag protein, mutations disrupting Gag multimerization, such as whole deletion of the MHR or point mutations of invariant residues within this region (CA residues 153–172), cause a reduction in Gag binding to membranes in vitro and in cell homogenates [132,133]. Similarly, mutations and/or truncations disrupting the CA dimer interface (CA W184A, M185A) [134,135], the helix contributing to the CA-SP1 six-helix bundle (CA L232A and SP1 M4A) [136,137], or NC-RNA-mediated multimerization (such as NC deletion) [138,139,140] disrupt the strong membrane binding of Gag. The proportion of Gag bound to the plasma membrane is correlated with its cellular concentration, with Gag at low concentrations remaining primarily cytosolic while higher concentrations, which presumably allow for greater multimerization, drive Gag localization primarily to the plasma membrane [110,141]. Together, these results strongly suggest that Gag–Gag interactions facilitating multimerization play a role in modulating Gag association with the membrane.

## 8. Mechanisms of Multimerization-Dependent Enhancement of Membrane Binding

In agreement with the genetic evidence of multimerization-dependent enhancement of membrane binding, in vitro membrane binding assays demonstrate that myristoylated MA fused to CA show a greater affinity for acidic lipid-containing membranes than myr-MA alone [22]. Additionally, dimerization-competent HIV-1 CA-CTD enhances membrane binding of MA-CTD fusion proteins relative to dimerization-deficient MA-CTD [142]. Similarly, HIV-1 MA protein chimeras containing inducible dimerization (FKBP) or constitutive hexamerization (Ccmk4) domains show enhanced binding to membranes containing PS, PS and cholesterol, or PS and PI(4,5)P_2_ [42]. Multiple mechanisms may contribute to the multimerization-dependent enhancement of Gag membrane binding including increased membrane binding avidity of the Gag multimer, multimerization-triggered exposure of myristate, Gag assembly mediated remodeling of the local plasma membrane microenvironment, and multimerization-triggered displacement of tRNA from the MA HBR.

### 8.1. Avidity

In early studies demonstrating that mutations disrupting NC-RNA-mediated multimerization also reduce HIV-1 Gag membrane binding [138,139], the authors proposed a model in which complexes of multimerized MA have a higher avidity for membranes due to clustered membrane binding domains. Although it lacks a myristate moiety, RSV MA has a patch of basic amino acids, which mediates membrane binding through interactions with acidic phospholipid head groups [143,144]. Relative to non-multimerizing constructs, which bind membrane poorly, dimerization-competent RSV proteins or chimeras containing RSV MA fused with strongly dimerizing HIV-1 CA-CTD (Q192A) show increased membrane binding [145]. A similar effect is seen when the membrane binding of RSV chimeras containing inducible dimerization (FKBP) or constitutive hexamerization (Ccmk4) domains is tested. In these experiments, dimerized or hexamerized MA demonstrates stronger binding to multiple membrane types [42]. Although myristate plays an important role in HIV-1 MA membrane binding, forced dimerization of non-myristoylated HIV-1 MA increases its affinity for binding membranes compared with monomeric MA that is either myristoylated or non-myristoylated [142]. Together, these experimental data support the model described earlier in which multimerization increases the avidity of Gag membrane binding through increased electrostatic interactions of clustered basic patches with acidic phospholipids.

### 8.2. Myristoyl Switch

A myristoyl switch model has been proposed to explain the differential binding of MA and full length Pr55Gag to membranes where, in the context of the isolated MA domain, myristate is sequestered within the MA globular head, but in the context of Pr55Gag, it is exposed to facilitate membrane binding [5,11,26,146,147,148,149]. More specifically, myristate exposure depends on the multimerization state of the MA domain; the myristate moiety is primarily sequestered within the MA hydrophobic pocket of monomeric MA but primarily exposed when MA is trimerized [128]. These studies agree with a model where Pr55Gag multimerization mediated by CA and NC domains stabilizes the MA-trimer interactions, leading to the exposure of myristate and priming MA for stable membrane binding.

### 8.3. Membrane Domain Reorganization

It has been recognized for some time that the lipid composition of the envelope of HIV-1 is different from the host cell plasma membrane from which it was derived. Compared with the lipid composition of the producer cell plasma membrane, HIV-1 particles are particularly enriched in specific lipids including PI(4,5)P_2_ and cholesterol [150,151,152]. While the profile of enriched membrane components was not identical between these studies, they supported the possibility that Gag membrane binding and viral assembly occur at specific microdomains of the plasma membrane, which would likely be compositionally distinct from the rest of the host cell plasma membrane [153,154,155,156]. While these studies are consistent with preferential Gag binding at potentially pre-existing PI(4,5)P_2_-enriched microdomains, more recent work has suggested that the multimerization of the Gag protein can re-model the membrane composition at the nascent site of assembly, thereby potentially driving the de novo formation of membrane micro-environments enriched in membrane components promoting further recruitment of Gag protein. Gag assembly has been shown to remodel the local membrane environment by driving the fusion of pre-existing microdomains, including lipid rafts and tetraspanin-enriched microdomains [157,158]. In addition to the remodeling of existing membrane domains, a study using an in vitro model membrane system [159] showed that Gag multimerization can drive the formation of membrane nanoclusters, which are enriched in both PI(4,5)P_2_ and cholesterol and display reduced mobility compared to free membrane lipids. A similar observation has been reported in cells, where Gag multimerization specifically traps PI(4,5)P_2_ and cholesterol within the site of assembly, while allowing for the free diffusion of non-enriched membrane components [160]. Recent in vitro membrane binding and fluorescent lipid quenching experiments further demonstrate that the Gag mutants, which lack an intact PI(4,5)P_2_ binding site (MA K29E, K31E) or efficient multimerization (CA W184A, M185A) fail to cluster PI(4,5)P_2_ [161]. This study also demonstrated that in contrast to the cellular PI(4,5)P_2_-specific membrane binding domain PH, which preferentially binds membranes containing freely diffusing PI(4,5)P_2_, myristoylated HIV-1 MA preferentially binds to membranes containing clustered PI(4,5)P_2_. Together, these results suggest a model in which Gag oligomerization drives the formation of its own micro-domain enriched in PI(4,5)P_2_ and cholesterol. Given that both PI(4,5)P_2_ (described above) and cholesterol [162,163] promote Gag membrane binding, the Gag multimerization-mediated enrichment of these membrane components in the local environment likely accelerates the recruitment of additional Gag molecules to the growing site of assembly.

### 8.4. Dissociation of tRNA

It is still unclear whether or how Gag oligomerization contributes to RNA-mediated regulation of Gag membrane binding. In the presence of tRNA, full length Gag retains the ability to bind membranes containing PI(4,5)P_2_ [34]. Purified MA, which lacks the capacity to multimerize through CA and NC, is not able to overcome tRNA-mediated inhibition of membrane binding even in the presence of PI(4,5)P_2_-containing membranes [52]. A recent co-crystal of MA-tRNA^Lys3^ complex suggests that tRNA could freely interact with MA domains in a trimer orientation but would be sterically prohibited from binding to the HBR of MA involved in higher-order oligomers [54]. Therefore, it is conceivable that Gag oligomerization, driven by CA and NC domains downstream of MA, promotes membrane binding in part by promoting the steric displacement of tRNA from MA HBR, priming some MA domains within a Gag complex for membrane binding.

## 9. Summary and Open Questions

Specific localization of HIV-1 Gag to the plasma membrane during viral assembly is primarily driven by specific binding to PI(4,5)P_2_ at the plasma membrane, paired with inhibition of non-PI(4,5)P_2_-containing membrane binding by tRNA. During assembly, Gag oligomerization is driven by inter-protein interactions between adjacent CA domains as well as scaffolding via NC-RNA and MA-membrane interactions. Gag multimerizes primarily on the plasma membrane, and lattice growth occurs through the addition of monomeric or dimeric Gag to the lattice edges from the cytosolic pool. In addition to its primary role in lattice assembly, Gag multimerization through CA-CA and NC-RNA interactions also promotes membrane binding via the MA domain. Multimerization-mediated enhancement presumably likely occurs through a combination of the following mechanisms: increase in membrane binding domain avidity, myristate exposure, remodeling of the local membrane microenvironment, and steric displacement of tRNA from the MA HBR.

There are still many remaining questions about mechanisms by which multimerization enhances the membrane binding of Gag and the implications of this effect for its trafficking and assembly. As mentioned above, it is unclear if Gag multimerization impacts RNA binding to the MA HBR. Whether tRNA is indeed removed upon Gag multimerization and whether the presence of PI(4,5)P_2_ in the membrane also affects the dependence of Gag membrane binding on multimerization need to be tested. If tRNA is displaced from MA-HBR upon Gag multimerization, this mechanism likely contributes to the temporal/spatial regulation of Gag membrane binding and the growth of the Gag lattice in two major ways: (1) enhancing efficient incorporation of Gag into active assembly sites by preventing non-productive membrane binding of monomeric or dimeric Gag and (2) priming cytosolic Gag complexes for membrane binding by reducing tRNA imposed inhibition. Related to the latter, evidence suggests that Gag-gRNA complexes seed lattice and particle assembly more efficiently than Gag multimer lacking gRNA. How do the characteristics of Gag multimers change when they contain gRNA? Do Gag multimers containing gRNA bind more efficiently to the plasma membrane than Gag multimers not associated with gRNA, which in turn would ensure gRNA packaging? Do gRNA-associated Gag clusters bound to the plasma membrane recruit cytosolic Gag more efficiently than those without gRNA? The enrichment of membrane components such as PI(4,5)P_2_ and cholesterol is observed at sites of Gag multimerization. How does this membrane remodeling contribute to viral assembly? Does the enrichment of PI(4,5)P_2_ or cholesterol promote recruitment of Gag preferentially to the site where Gag multimer pre-exists, thereby facilitating growth of the Gag lattice? Does the remodeling of the local membrane environment at the site of assembly regulate the incorporation of host-derived co-factors into the assembling virus particle? Addressing these questions will undoubtedly advance our understanding of the process of Gag membrane binding and multimerization and will contribute to our understanding of the mechanisms promoting the productive assembly of HIV-1 particles specifically at the plasma membrane of infected cells.

## Data Availability

Not applicable.
